# GASZ directly recruits MILI to the intermitochondrial cement for piRNA biogenesis and male germ cell development

**DOI:** 10.1093/nar/gkaf957

**Published:** 2025-10-08

**Authors:** Junru Miao, Zhaoran Zhang, Duong Nguyen, Hanben Wang, Danella Gong, Maddison Marshall, Yinjiao Xu, Huirong Xie, Chuanyun Wang, Jingjing Zhang, Yongsheng Wang, Yuan Wang

**Affiliations:** Department of Animal Sciences, College of Agriculture and Natural Resources, Michigan State University, East Lansing, MI48824, United States; Department of Animal Sciences, College of Agriculture and Natural Resources, Michigan State University, East Lansing, MI48824, United States; Department of Animal Sciences, College of Agriculture and Natural Resources, Michigan State University, East Lansing, MI48824, United States; Department of Animal Sciences, College of Agriculture and Natural Resources, Michigan State University, East Lansing, MI48824, United States; Department of Animal Sciences, College of Agriculture and Natural Resources, Michigan State University, East Lansing, MI48824, United States; Department of Animal Sciences, College of Agriculture and Natural Resources, Michigan State University, East Lansing, MI48824, United States; Department of Animal Sciences, College of Agriculture and Natural Resources, Michigan State University, East Lansing, MI48824, United States; Transgenic and Genome Editing Facility, Institute for Quantitative Health Science & Engineering, Michigan State University, East Lansing, MI 48824, United States; Shanghai Key Laboratory of Regulatory Biology, Institute of Biomedical Sciences and School of Life Sciences, East China Normal University, Shanghai, 200241, China; Translational Medicine Research Center, Affiliated Hangzhou First People's Hospital, School of Medicine, Westlake University, Hangzhou, Zhejiang, 310006, China; Shanghai Key Laboratory of Regulatory Biology, Institute of Biomedical Sciences and School of Life Sciences, East China Normal University, Shanghai, 200241, China; Department of Animal Sciences, College of Agriculture and Natural Resources, Michigan State University, East Lansing, MI48824, United States

## Abstract

Repressing transposable elements *via* piRNAs represents a critical defense mechanism for germ cells to maintain genomic integrity. The primary piRNA biogenesis largely occurs at intermitochondrial cement (IMC), which is characterized by uniquely clustered mitochondria and ribonucleoproteins as “cementing material.” RNA-binding proteins at IMC, such as MILI, are essential for piRNA biogenesis. However, MILI proteins do not possess mitochondrial localization signals; thus, they must rely on other proteins to functionally communicate with IMC. In this study, we identified GASZ as a crucial interacting partner for MILI at IMC from prospermatogonia to spermatocytes. We found that GASZ proteins at mitochondria directly recruited MILI to IMC for piRNA biogenesis. Abolishing GASZ–MILI interaction in the embryonic germ cells reduced fetal piRNA level, increased transposon expression, and compromised spermatogonial and spermatocyte development during the first wave of spermatogenesis. In addition, disrupting GASZ–MILI interaction in adulthood significantly impaired spermatogenesis, with reduced spermatocyte and spermatid formation, proving that MILI and GASZ partner together to regulate steady-state spermatogenesis. Taken together, by revealing critical GASZ–MILI interaction at IMC and defining its impact on spermatogenesis, our findings critically inform how the piRNA biogenesis machinery is constructed *via* protein interactions to preserve germline DNA integrity for proper germ cell development.

## Graphical Abstract

**Graphical Abstract:**
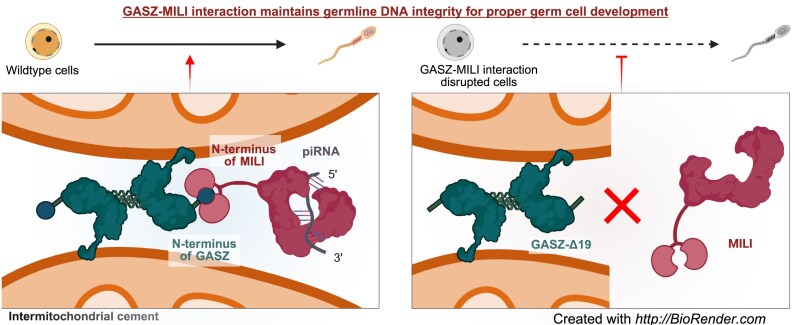

## Background

It is crucial for germ cells to pass the correct genetic information through generations. P-element-induced wimpy testis (PIWI)-interacting RNAs (piRNAs) play a critical role during this process by silencing transposons to maintain germline DNA integrity [1–5]. Mature piRNAs are small non-coding RNAs with ∼24–34 nucleotides in length and have been identified in germ cells of diverse species [1–5]. During male germ cell development, piRNA biogenesis starts when the bi-potential primordial germ cells become prospermatogonia (also called gonocytes) in embryonic gonads. After birth, some of the prospermatogonia develop into spermatogonial stem cells (SSCs) to sustain spermatogenesis, during which primary spermatocytes from differentiating spermatogonia undergo a long meiotic prophase I, passing through the stages of leptotene, zygotene, pachytene, diplotene, and diakinesis, followed by two chromosome divisions to finally produce haploid spermatids [6, 7]. The piRNAs are expressed at a relatively high level in prospermatogonia during embryonic stages and in neonatal testes (*i.e*. fetal piRNAs). Their level then drops in postnatal spermatogonia (*i.e*. pre-pachytene) and becomes abundant again in pachytene spermatocytes and spermatids (*i.e*. pachytene piRNAs) [1–5, 8]. Fetal piRNAs are largely derived from transposable elements, with a small fraction (<20%) from exons of protein-coding regions [9]. These piRNAs in turn repress the transcription of transposable elements, a function essential for proper germ cell development [1–5], while pachytene piRNAs display enormous diversity in their sequences and may also participate in post-transcriptional regulation [10–13].

The piRNA biogenesis in male germ cells largely occurs at nuage, the amorphous electron-dense granules in the cytoplasm that can be observed with transmission electron microscopy (TEM) [14–17]. Based on the size, subcellular location, and components, nuage is classified as intermitochondrial cement (IMC, also known as pi-body), piP-body, and chromatoid body [17, 18]. The IMC is present in prospermatogonia, spermatogonia, and pachytene spermatocytes. At IMC, clustered mitochondria with ribonucleoproteins as “cementing materials” are uniquely present [17, 18]. Disrupting IMC formation or removing RNA-binding proteins from IMC often reduces piRNA biogenesis, blocks spermatogenesis, and impairs male fertility [19–27].

PIWI proteins are the main players in piRNA biogenesis [19, 20, 23, 28]. They belong to the PIWI/Argonaute family and are evolutionarily conserved with the presence of PAZ (Piwi-Argonaute-Zwille) and PIWI domains [19, 20, 23, 28]. PIWI proteins are predominantly expressed in germ cells, playing indispensable roles in spermatogenesis. Three PIWI members, MIWI (*i.e*. PIWIL1), MILI (*i.e*. PIWIL2), and MIWI2 (*i.e*. PIWIL4), have been identified in mice [19, 20, 28, 29]. Among them, MIWI2 largely localizes the nucleus of prospermatogonia and acts as a direct effector with piRNAs in the methylation of transposable elements [9, 30]. MIWI2 also participates in secondary piRNA processing at piP-bodies, while both MILI and MIWI predominantly localize at IMC and chromatoid body for piRNA processing [9, 30]. MILI is particularly interesting because its expression starts in embryonic prospermatogonia and persists after birth into the spermatid stage [9, 30]. MILI appears to regulate the biogenesis of both pre-pachytene and pachytene piRNAs, while MIWI contributes to pachytene piRNA processing [9, 30]. In addition to PIWI family members, several RNA-binding proteins also participate in piRNA biogenesis at IMC, including TDRD1 [22] and DDX4 [24]. None of these proteins possesses a mitochondrial localization signal (MLS). It remains puzzling how these proteins are recruited to or functionally communicate with IMC.

GASZ (*aka*. ASZ1) is a **G**erm cell-specific protein with four **A**nkyrin repeats/ANK, a **S**terile alpha motif/SAM, and one leucine **Z**ipper/ZIP. GASZ is localized at the mitochondrial outer membrane via *C*-terminal MLS [25, 26, 31]. We previously found that the deletion of MLS from GASZ dislocated it from mitochondria to the cytoplasm of germ cells [26]. Consequently, no IMC was formed, piRNA expression was drastically reduced, and spermatogenesis was arrested at the spermatocyte stage [26]. Like MILI, GASZ starts to express around embryonic day (**E**) 13 when IMC is initially formed [32]. In this study, we revealed GASZ as a critical partner for MILI at mitochondria. GASZ directly recruited MILI to IMC via its *N*-terminal 20 amino acids in germ cells. Abolishing GASZ–MILI interaction during the embryonic stage reduced spermatogonial development, and no spermatids formed when this occurred in adult mice.

## Materials and methods

### GASZ mutant mice and viral injection

All animal experimental procedures were performed according to the protocol (PROTO202300165) approved by the Institutional Animal Care and Use Committee at Michigan State University. *Gasz* deletion mutant founder mice were generated at the MSU transgenic core facility *via* CRISPR/CAS9 gene editing. Founder mice with correct deletion were identified by Sanger sequencing of *Gasz* genomic locus and then crossed with C57BL/6 mice (000 664; The Jackson Laboratory) to get heterozygote *Gasz^+/Δ19^* mice. *Gasz^Δ19/Δ19^*were obtained by crossing heterozygote *Gasz^+/Δ19^* mice, or by crossing *Gasz^+/Δ19^* male with *Gasz^Δ19/Δ19^*female mice. The sgRNAs and oligonucleotide template to generate *Gasz* deletion mutant founder mice, as well as genotyping PCR primers were listed in Supplementary Table S4. Lentiviral injection *via* the efferent duct into testis was performed as described previously [33]. Briefly, approximately 5–10 μL of lentivirus with trypan blue were injected into the seminiferous tubules of 5-week-old C57BL/6 mice (the Jackson Lab). Testes were analyzed at 8 weeks post viral injection.

### Histology study and IHF assays

For histology analyses, testes were fixed in Bouin’s solution (Sigma-Aldrich, HT10132), followed by dehydration, embedding in paraffin, and cut into 4 μm sections for hematoxylin (Sigma-Aldrich, MHS1) and eosin (Sigma-Aldrich, E4009) staining. Permount (SP15100; Fisher Scientific) was applied onto the sections. The sections were then covered by coverslips and dried in the hood overnight before imaging. For IHF staining, 4 μm testis sections were permeabilized with PBS containing 0.4% Triton X-100 and washed with PBS. 10 mM sodium citrate buffer (pH7.4) was used for antigen retrieval. Sections were blocked with blocking buffer (5% BSA in PBS) incubated with primary antibodies and secondary antibodies, followed with a drop of Glass Antifade Mountant (P36982; Invitrogen) for imaging. Antibodies were used in this study below: FLAG (Sigma-Aldrich, F7425 & F1804; Abmart, M20088), HA (Abmart, M20088), GASZ (Thermo Fisher, 21550–1-AP), MILI (Cell signaling, 5940), PRM1 (Briar Patch Biosciences, MAb-Hup1N-150), Antibodies from Abcam: DDX4 (ab196708 and ab13840), SYCP3 (ab15093 and ab97672), TRA98 (ab82527), TOMM20 (ab186734). Antibodies from Invitrogen: Donkey anti-Mouse Alexa Fluor 488 (A21202), Donkey anti-Rabbit Alexa Fluor 488 (A32790TR), Donkey anti-Mouse Alexa Fluor 555 (A31570), Donkey anti-Rat Alexa Fluor 555 (A78945), Donkey anti-Goat Alexa Fluor 555 (A21432;), and Donkey anti-Rabbit Alexa Fluor 647 (A-31573). Antibodies from Jackson ImmunoResearch: Goat anti-Mouse Alexa Fluor 594 (115–585–003), Goat anti-Rabbit Alexa Fluor 594 (111–585–003). Microscopes were used in this study: Olympus FL confocal microscope, Nikon Eclipse Ti microscope, BioTek Lionheart FX Automated Microscope, Leica DM4000B microscope, Nikon A1 CLSM microscope, or Leica TCS/SP5 confocal microscope.

### In situ proximity ligation assays

Proximity ligation assay (PLA) was processed by using Duolink *in aitu* reagents according to manufacturer's manual. Briefly, deparaffinization, rehydration and antigen retrieval of testis sections were performed. Testis sections were incubated with primary antibodies followed by washing three times with PBS containing 0.2% Tween-20. Duolink *in situ* probes containing PLUS (Sigma, DUO92002-30RXN) and MINUS (Sigma, DUO92004-30RXN) were used to incubate with sections at a pre-heated humidity chamber for 1 h at 37°C. Samples were subjected to ligation and rolling circle amplification (Sigma, UO92008-30RXN) and mounted with Duolink® In Situ Mounting Medium (Sigma, UO82040-5ML). Images were captured with Olympus microscopes. Primary antibodies were used in this assay were anti-MILI (Santa Cruz, sc-377258) and anti-GASZ (Thermo Fisher, 21550–1-AP).

### Transmission electron microscopy

TEM was performed according to published protocols [34]. Briefly, P3 mouse testes were collected and fixed with 2.5% TEM-grade glutaraldehyde in 0.1 M cacodylate buffer (AAJ60367AE; Thermo Scientific Chemicals). Samples were washed with 0.1 M cacodylate buffer and postfixed with 1% osmium tetroxide in 0.1 M cacodylate buffer. After fixation, samples were dehydrated in a gradient series of acetone, infiltrated, and embedded in Spurr resin. Specimen blocks were trimmed and cut into 100 nm ultra-thin sections. These ultra-thin sections were collected onto a 200-nm grid and stained with 1% uranyl acetate, followed by staining with lead citrate. TEM images were acquired with ultra-high resolution JEOL 2200FS electron microscope at 80 kV.

### Plasmid construction for expressing transgenes and peptides

*Gasz* cDNA and Su9-GFP plasmid were described in our previous study [26]. *Mili, Miwi, Ranbp9*, and *Ddx4* expression plasmids were kind gifts from Dr. Michael A. Frohman at Stony Brook University. *Mili, Miwi*, and *Ddx4* were sub-cloned into pll3.7 fused with a FLAG tag, while *Ranbp9* was subcloned into *pDsRed-C1* in frame with DsRed. *Gasz* cDNA and *Gasz* mutants were cloned into the following vectors with its *N*-terminus fused with EGFP in *pEGFP-c1*, FLAG in *pll3.7*, or GST in *pGEX-4T-1*. P20, the GASZ–MILI interaction blocking peptide, was generated by PCR and fused with FLAG in *pll3.7*. *Mili* cDNA was cloned into *pEGFP-c1* and *pET17* with its *N*-terminus fused with GFP or a HIS tag respectively, or into *pcDNA3.1* with its C-terminus fused with a HA tag. MILI deletion mutants were generated by PCR and cloned into *pll3.7* with its *N*-terminus in frame with FLAG. All plasmids were sequenced to verify the correct inserts and mutations. Primers for cloning and mutagenesis are listed in Supplementary Table S4.

### Cell culture, transfection, and viral infection

HeLa and 293T cells were cultured in DMEM (Gibco) with 10% fetal bovine serum (Gibco) and transfected with plasmids using polyethylenimine (Sigma) for ectopic gene expression. Mouse spermatogonia were cultured according to published protocols [35, 36]. For viral infection of spermatogonia, lentivirus was packaged in 293T cells and concentrated by Amicon® Ultra-50 Centrifugal Filter Unit (Sigma, UFC9100) into 6 × 10^7^/ml. Cell suspension was mixed with concentrated virus with MOI at 2 and 5 μg/mL polybrene. Cell medium was changed after 20 h post-infection.

### Immunofluorescence (IF) assays

Cells were seeded on cover slides and fixed in 4% paraformaldehyde for 30 min at room temperature. Following permeabilization in 0.04% Triton X-100, IF was performed using the following antibodies: HA antibody (Abmart, MA9028), GFP (Abcam, ab183734), FLAG (Sigma, F1804), GASZ (generated from rabbits using GST-GASZ as an immunogen), MILI (Cell Signaling Technology, 5940S) in 5% BSA/PBS. The fluorochrome-conjugated 2^nd^ antibodies (Jackson ImmunoResearch Inc. & Invitrogen) were used with 1:200 dilution. For mitochondrial staining, live cells were incubated with MitoTracker Red FM (ThermoFisher, M7512, 1:5 000 dilution) at 37°C in dark for half an hour before fixation for IF. Images were acquired using a Leica TCS/SP5 confocal microscope or a Nikon A1 CLSM microscope.

### Western Blot, Co-IP, and HIS-tagged protein pull-down assay

Western blots were performed according to standard protocols. Primary antibodies were used in this study: FLAG (Abmart, M20088; Sigma, F7425), HA (Abmart, MA9028), GFP (Abcam, ab183734, Yeasen, 31001ES50), MILI (Cell Signaling Technology, 5940S), GASZ (generated from rabbits using GST-tagged full-length GASZ as an immunogen). The specificity of home-made GASZ antibody was tested by Western Blots and IF on GASZ overexpressing cells (Supplementary Fig. S6D & E). The fluorochrome-conjugated 2^nd^ antibodies were used at 1:10 000 dilution (LI-COR Biosciences, P/N: 926–49 020; LI-COR Biosciences P/N: 926–68 070). The fluorescent signals against targeted proteins were detected using the Li-COR Odyssey system. For co-immunoprecipitation (Co-IP), immunoprecipitation was performed as described previously [26]. Briefly, cells were harvested in lysis buffer (50 mM Tris, pH7.6, 300 mM NaCl, and 1% Triton X-100 with protease inhibitor cocktail) and sonicated at 4°C. Cleared supernatant was incubated with FLAG affinity beads (Sigma, A2220), HisPur™ Ni-NTA Resin (ThermoFisher Scientific, 88 221) or Protein A/G Agarose (Santa Cruz Biotech, sc-2003) incubated with IgG, GASZ, or MILI. The immunoprecipitated proteins were detected using Western blot. For protein pull-down by HIS-tagged MILI, GST‐GASZ fusion protein was induced by IPTG in bacteria at 30°C overnight, purified, and immobilized onto glutathione agarose beads. The protein–bead mixture was incubated with thrombin, which has specific proteolytic activity to cleave arginine-glycine peptide bonds between GST and GASZ to release GASZ. HIS-MILI fusion protein was induced by IPTG in bacteria at 30°C overnight and then incubated with purified GASZ at 4°C overnight and collected by Ni-NTA. The bead‐bound proteins were washed five times with PBS containing 1% Triton X‐100 before being separated by SDS–PAGE and detected by Western blots.

### PCR and real-time RT-PCR

Total RNAs were extracted with TRIzol (ThermoFisher), and 0.5–3 μg total RNAs were reverse-transcribed using a PrimeScrip RT reagent Kit (TAKARA, RR037A) according to manufacturers’ instructions. PCR and real-time PCR were performed as described previously [26]. Briefly, real-time PCR was performed with SYBR Premix Ex Taq (TAKARA) on Mx3005P (Stratagene) or a Quant Studio 5 PCR machine (Applied Biosystems). The transcript levels of transposon elements and piRNA were normalized to glyceraldehyde 3-phosphate dehydrogenase (GADPH) and 5S rRNA respectively. Primers used in this study were listed in Supplementary Table S4.

### RNA immunoprecipitation (RIP), small non-coding RNA-sequencing

Mouse testes were collected and homogenized using RIP lysis buffer (25 mM This-HCl pH 7.4, 150 mM NaCl, 0.2% NP-40, and 1 mM DTT) with protease inhibitor (04 693 132 001; Roche) and RNase inhibitor (n8080119; Applied Biosystems). The lysates were centrifuged at 14 000 rpm for 10 min to remove the debris, and the supernatant was pre-cleared using Protein A/G Magnetic Beads (B23201, Selleckchem). MILI (PM044, MBL) antibody and the Protein A/G Magnetic Beads were incubated at 4°C for 4 h and he mixture was added into pre-cleared cell lysates for incubation at 4°C for 4 h. The beads were washed in RIP lysis buffer five times. Immunoprecipitated RNAs were eluted from the beads using SPLIT RNA Extraction Kit (008.48, Lexogen) following the manufacturer's manual. Enriched small RNAs by SPLIT RNA Extraction Kit or small RNAs pulled down by RIP were subjected to library construction using Lexogen Small RNA-Seq Library Prep Kit. For detection of piRNA in cultured germ cells, primary spermatogonia expressing either the control FLAG or FLAG-P20 peptides were collected for constructing small RNA libraries with GenSeq® Small RNA Library Prep Kit according to manufacturer's instruction (GenSeq, Inc.). Briefly, 3′ and 5′ adaptors were successively ligated to RNAs, which were reversely transcribed into cDNA, followed by PCR amplification. The barcoded cDNA libraries were size selected for piRNA fractions (20–40 nt) before sequencing with a NovaSeq platform. The adaptor sequences were trimmed from raw data using a cutadapt software, and the trimmed sequences (≥15nt) were aligned to known piRNAs from piRNABank using the Novoalign software (v3.02.12) allowing for one mismatch or less. The read counts per million aligned piRNAs were normalized to miRNA counts (21–23 nt). The piRNA targets were predicted using miranda software (v3.3a). The 5′ nucleotide preference [37] and size distribution [24] of piRNAs were calculated according to published protocols. To calculate the 5′-5′ end distances, piRNA reads were mapped on the opposite strand within a 7–20 nt window. Only uniquely mapped reads were included. The z-score for 10 nt overlap (ping-pong amplification signature) was calculated using the following formula [38]: $Z = \frac{{\omega ( {0 = 10} ) - mean( {\omega ( {0 \ne 10} )} )}}{{\sqrt {var( {\omega ( {0 \ne 10} )} )/n} }}$

### AlphaFold 2 prediction of protein structure and protein interactions

The full-length protein sequences of mouse GASZ (NP_076218.3) and MILI (NP_076218.3) were downloaded from NCBI. Structures of GASZ and GASZ-MILI were predicted using the AlphaFold 2 at Google Colab followed the published protocol [39]. Models were generated with the following parameters: rank number = 5, multiple sequence alignment mode = MMseqs2, pair_mode = Unpaired + paired, and number_recycles = 3. The top five models were ranked based on predicted local distance difference test scores per-residue. These models were analyzed by PyMOL software for visualization of the interacting interface. All top five models showed the similar structure of GASZ protein and interface of *N*-terminal (aa 1–30) of GASZ interacting with MILI.

### Statistical analysis

Data were presented as mean ± SEM. All experiments were performed independently three times or more unless otherwise stated. Statistical comparisons of between-group means were performed using unpaired Student's t test and the Prism GraphPad software.

## Results

### GASZ directly recruits MILI to mitochondria in germ cells

**Figure 1.**
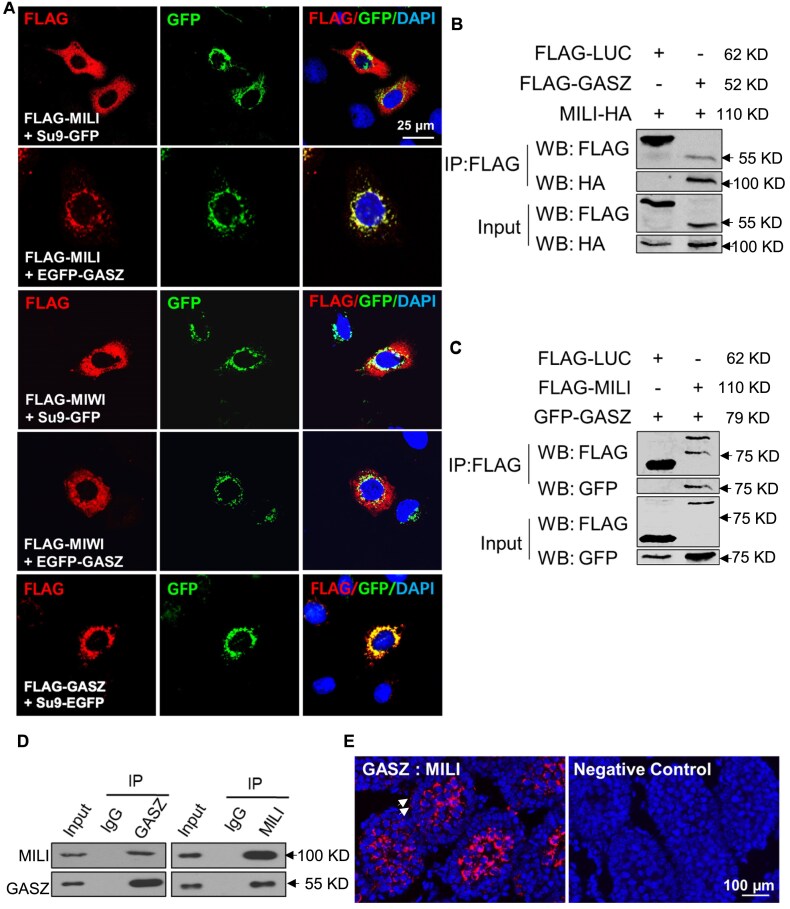
GASZ directly recruits MILI to mitochondria. (**A**) FLAG-tagged MILI was co-expressed with either Su9 MLS-tagged GFP or EGFP-GASZ fusion proteins in HeLa cells. Protein subcellular localization was visualized by IF using antibodies against GFP (green) and FLAG (red), counterstained with a DNA dye DAPI (blue). Cytoplasmic MILI was recruited by GASZ to colocalize with GASZ at mitochondria. (**B**) HA-tagged MILI was co-expressed with FLAG-tagged GASZ in 293T cells. (**C**) FLAG-tagged MILI was co-expressed with GFP-tagged GASZ in 293T cells. The top blot was not completely stripped off and thus showed both proteins (79 KD versus 110 KD). (**B**-**C**) Cell lysates were immunoprecipitated (IP) with FLAG affinity beads, followed by Western Blot (WB) analyses with antibodies listed on the left. FLAG-Luciferase (FLAG-LUC: 62 KD) was used as a control. (**D**) Co-IP with antibodies against either MILI (110 KD) or GASZ (52 KD) on adult mouse testes. Antibodies used for Western blots are shown on the left. (**E**) PLA assay on mouse testes at P14 with MILI and GASZ antibodies. Red signals indicate interaction between MILI and GASZ. A negative control using GASZ antibody alone was shown on the right. Spermatocytes are cells in the middle of the seminiferous tubules. White arrows indicate spermatogonia. (**B**-**D**) The molecular weights (MW) of the proteins to be detected are provided on the right side of the panels. Arrows indicate the position of the protein ladders with known MW.

We previously revealed that upon dislocation of GASZ proteins from mitochondria in germ cells, IMC proteins (e.g. MILI and DDX4) displayed a diffused cytoplasmic localization, with MILI entirely into cytoplasm [26]. These data lead us to hypothesize that GASZ directly recruits these proteins to mitochondria within IMC or indirectly *via* interacting with other protein partners. To distinguish these two possibilities, we examined the interaction of GASZ with MIWI, MILI, DDX4, and RANBP9 by co-expressing these proteins with GASZ in HeLa cells in the absence of other germ cell-specific proteins. Consistent with previous findings, we observed that GASZ was entirely localized to mitochondria marked by GFP tagged with Su9 MLS (Fig. 1A), a commonly used mitochondrial targeting sequence derived from subunit 9 of the Neurospora Crassa ATPase [26]. MILI displayed a cytoplasmic localization when GASZ was absent but was readily recruited to mitochondria when they were co-expressed, examined by immunofluorescence (IF) (Fig. 1A & Supplementary Fig. S1A and B). By contrast, the subcellular localization of other proteins, such as MIWI, DDX4 and RANBP9, was not altered when the mitochondrially localized GASZ was present (Fig. 1A & Supplementary Fig. S1A–C), suggesting that GASZ only interacts with MILI directly but not with other IMC proteins. We confirmed these findings in 293T cells, another type of somatic cells that lack other germ cell-specific proteins. MILI and GASZ proteins were reciprocally co-immunoprecipitated when co-expressed in 293T cells (Fig. 1B and C), while MIWI could not be pulled down with GASZ (Supplementary Fig. S1D). We further mixed purified GASZ proteins with HIS-tagged MILI, both of which were produced from bacteria. We found that MILI proteins retained by Ni-NTA, an HIS affinity column, could efficiently pull down GASZ from bacterial lysate (Supplementary Fig. S1E), supporting direct interaction between GASZ and MILI.

Similarly, endogenous MILI and GASZ interacted with each other in testes, examined by Co-IP (Fig. 1D). We further confirmed these results with an *in situ* Duolink PLA. In this assay, one pair of oligonucleotides are separately linked with two antibodies against target proteins (*i.e*. MILI and GASZ). When two target proteins interact in close proximity (<40 nm), these two oligonucleotides hybridize to form circular DNAs, which are further amplified and detected by complementary fluorescent oligonucleotide probes. We observed robust PLA signals in spermatocytes from mouse testis at postnatal day (P)14 (Fig. 1E). PLA signals were also observed in prospermatogonia at E16.5 (Supplementary Fig. S1E) and spermatogonia at P14 (Fig. 1E), though at relatively low levels. No signals were detected in negative controls when only one antibody was added (Fig. 1E & Supplementary Fig. S1F). Taken together, our data revealed the direct interaction of GASZ with MILI under the physiological setting and conditions upon their enforced co-expression in somatic cells.

### The *N*-terminal region of GASZ is essential for its interaction with MILI

**Figure 2.**
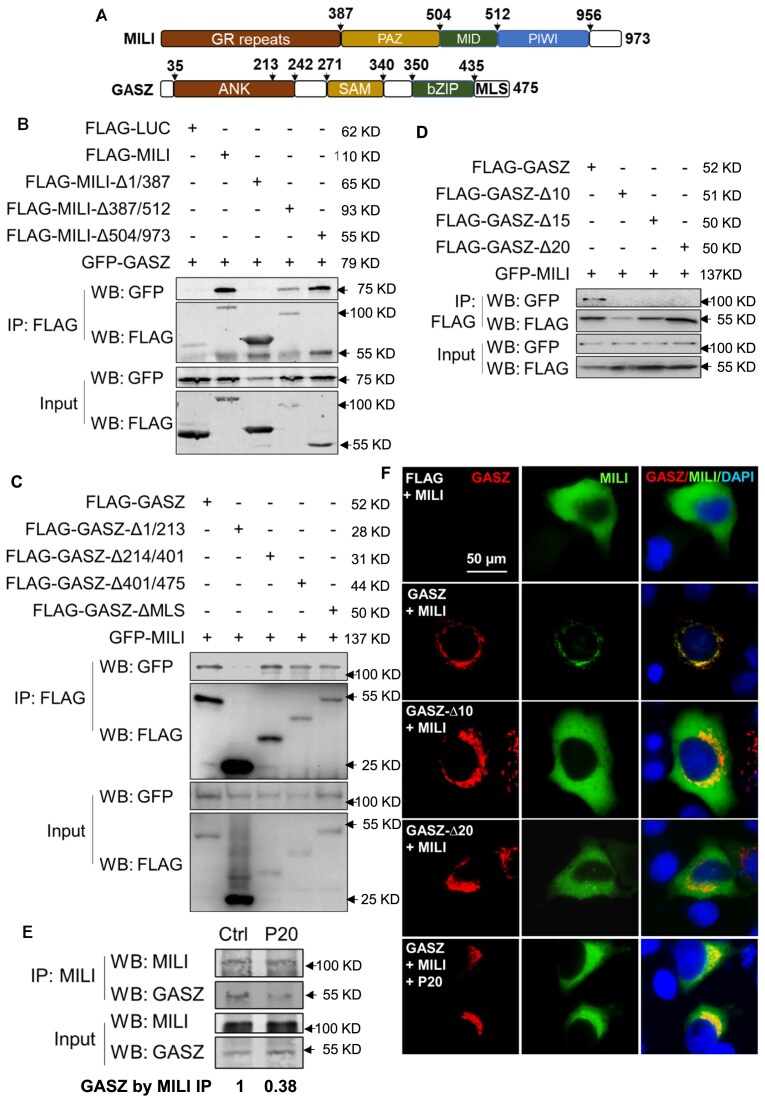
GASZ interacts with MILI via its *N*-terminal 20 aa. (**A**) Functional domains of GASZ and MILI proteins with beginning and ending aa annotated. (**B**) GFP-tagged GASZ proteins were co-expressed in 293T cells with FLAG-tagged full-length MILI or various MILI deletion (Δ) mutants, labeled with beginning and ending aa of deleted regions. (**C**) GFP-tagged MILI proteins were co-expressed in 293T cells with FLAG-tagged full-length GASZ or various GASZ mutants, annotated with beginning and ending aa of deleted regions. (**D**) GFP-tagged MILI proteins were co-expressed in 293T cells with either FLAG-tagged full-length GASZ or GASZ mutants with deletions from the 2^nd^ to the designated aa residue. (**B** **–D**) Cell lysates were immunoprecipitated (IP) with FLAG affinity beads for Western Blot (WB) analyses. FLAG-LUC: FLAG-Luciferase. MILI without GR repeats or GASZ without the first 20 amino acid residues failed to pull down each other. (**E**) FLAG (Ctrl) or FLAG-tagged peptide expressing the first 20 aa of GASZ protein (P20) was introduced into primarily cultured spermatogonia via lentiviral infection. MILI and GASZ interaction was examined by Co-IP with a MILI antibody, followed by Western Blot (WB) analyses. The numbers below the panel were fold changes of the levels of GASZ proteins co-precipitated with MILI in the presence of control FLAG or FLAG-P20 blocking peptide, normalized by input. (**F**) HA-tagged MILI was co-expressed with GASZ deletion mutants or full-length GASZ in the presence of FLAG-P20 (P20) or a FLAG control peptide in HeLa cells. Protein subcellular localization was visualized by IF using antibodies against HA (red) and GASZ (green), counterstained with DAPI (blue). P20 reduced MILI recruitment by GASZ to mitochondria. (**B**–**E**) The MW of the proteins to be detected are provided on the right side of the panels. Arrows indicate the position of the protein ladders with known MW.

We next sought to identify the critical regions that were responsible for GASZ–MILI interaction with Co-IP assays in 293T cells. When full-length GASZ was co-expressed with various MILI deletion mutants, we found that the *N*-terminal region of MILI containing Glycine/Arginine (GR) repeats was required for its interaction with GASZ (Fig. 2A and B). In addition, without its *N*-terminal amino acid (aa) residues from 1 to 213, GASZ could no longer interact with MILI (Fig. 2A and C). Further deletion mutagenesis discovered that the first 20 aa residues of GASZ were critical for its interaction with MILI (Fig. 2D & Supplementary Fig. S2A). The protein structures that were predicted by AlphaFold2 also supported the direct interaction of these GASZ aa residues with MILI (Supplementary Fig. S2B and C). We then developed a FLAG-tagged peptide containing only the first 20 aa (P20) of GASZ and introduced it into primarily cultured spermatogonia by lentivirus. We found that this peptide could competitively reduce the interaction between the endogenous MILI and GASZ proteins, examined by Co-IP followed by Western Blots (Fig. 2E and Supplementary Fig. S2D). We confirmed these results by IF in HeLa cells. In this assay, exogenously expressed HA-tagged MILI proteins predominantly localized in the cytoplasm but were readily recruited to mitochondria by full-length GASZ (Fig. 2F). This mitochondrial recruitment was abolished when MILI was co-expressed with mutant GASZ proteins lacking 2–10 or 2–20 aa (Fig. 2F). In the presence of the blocking peptide expressing the first 20 aa of GASZ, full-length GASZ was no longer able to recruit MILI to mitochondria (Fig. 2F). In summary, we identified the crucial domains within GASZ and MILI for GASZ–MILI interaction.

### Deletion of the *N*-terminal 19 aa of GASZ disrupts GASZ–MILI interaction in germ cells

To determine the functional consequence of disrupted GASZ–MILI interaction in germ cell development, we generated a GASZ mutant mouse model with its *N*-terminal 2 to 20 aa deleted via CRISPR-CAS9 gene editing technology (Supplementary Fig. S3A and B). Heterozygote GASZ mutant founder mice were bred with each other to obtain homozygote GASZ deletion mutants (*i.e*.*Gasz^Δ19/Δ19^*), in which the correct deletion of *N*-terminal 2–20 aa was confirmed by Sanger sequencing and PCR-based genotyping (Supplementary Fig. S3A and B).

**Figure 3.**
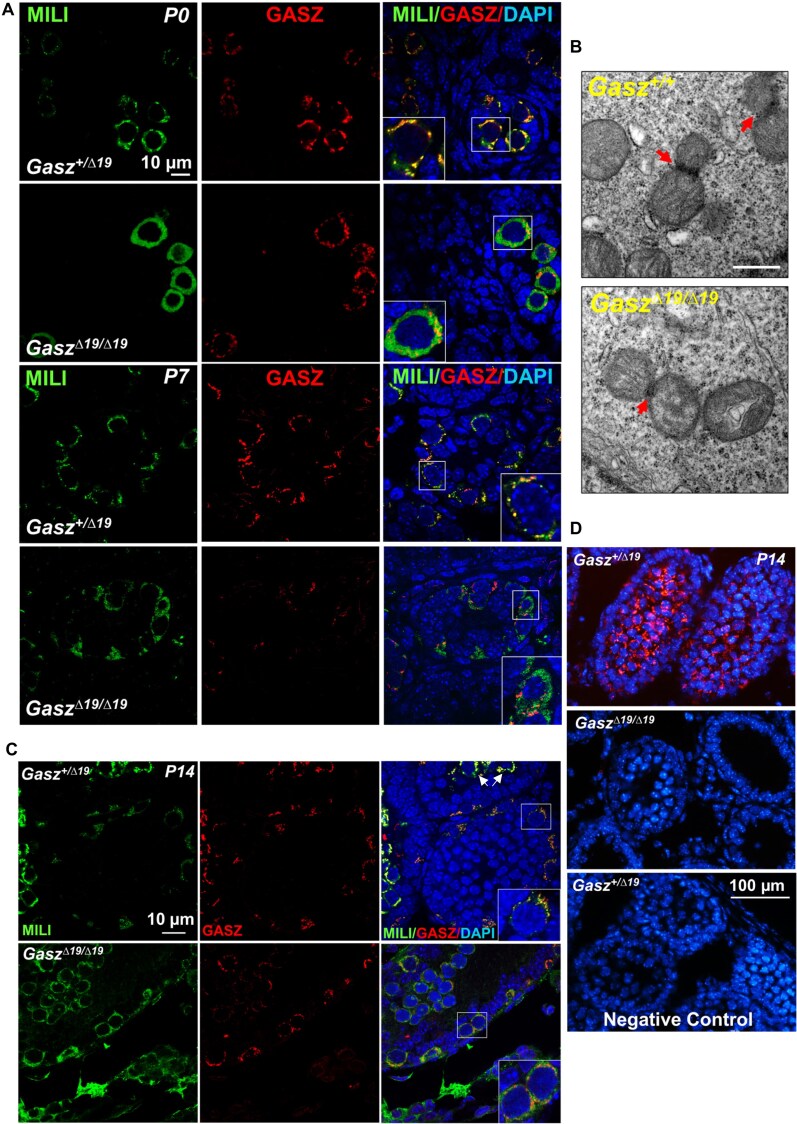
Establishing a mutant GASZ mouse model with disrupted GASZ–MILI interaction. (**A**) The localization of MILI and GASZ proteins in testes from neonatal (P0) or P7 *Gasz^Δ19/Δ19^*mice and heterozygote littermate controls (*Gasz^+/Δ19^*) was analyzed by IHF with antibodies against MILI (green) and GASZ (red), counterstained with DAPI (blue). MILI was diffused in *Gasz^Δ19/Δ19^*germ cells. (**B**) IMC structure was examined by TEM on testicular sections from *Gasz^Δ19/Δ19^* mice and their wildtype littermate controls at P3. Red arrows point to IMC in spermatogonia. (**C**) The localization of MILI and GASZ proteins in testes from P14 mice was analyzed by IHF with antibodies against MILI (green) and GASZ (red), counterstained with DAPI. The white arrows point to spermatocytes, while the insets show blowup images of spermatogonia. (**D**) PLA assay on mouse testes from *Gasz^Δ19/Δ19^* mice and their heterozygote littermate controls at P14. GASZ antibody alone was used as a negative control. (**A**, **C**) GASZ and MILI signals were dim in *Gasz^Δ19/Δ19^* germ cells from P7 and P14 mice. A relatively higher exposure was used in *Gasz^Δ19/Δ19^* photos compared to those in the *Gasz^+/Δ19^* control to visualize the subcellular localization of fluorescent signals.

Both GASZ and MILI start to express during the embryonic stage [32]. We thus examined whether the GASZ–MILI interaction in *Gasz^Δ19/Δ19^*mice was disrupted by immunohistofluorescence (IHF) in prospermatogonia from neonatal mice. Consistent with published findings [25, 26], we observed that MILI exhibited a granule distribution and largely co-localized with GASZ in control mice (Fig. 3A). By contrast, MILI diffused into the cytoplasm in *Gasz^Δ19/Δ19^*prospermatogonia, and no longer co-localized with GASZ signals (Fig. 3A). At P7, the expression levels of GASZ and MILI were both decreased in *Gasz^Δ19/Δ19^* testes. Nevertheless, compared to control spermatogonia, the granular localization pattern of GASZ proteins was not altered in homozygote GASZ mutant mice, while MILI signals were dispersed and rarely co-stained with GASZ (Fig. 3A). Signals of mitochondrial protein TOMM20 at P7 largely remained as patches and granules with slightly smaller sizes in *Gasz^Δ19/Δ19^*, compared to their littermate controls (Supplementary Fig. S3C). Consistently, the size of “cement” within IMC was reduced, but IMC could still be found in homozygote GASZ mutant germ cells (Fig. 3B), examined by TEM. These data reveal that although IMC partially remains, MILI proteins are dissociated from GASZ at the mitochondria and diffuse into the cytoplasm in *Gasz^Δ19/Δ19^*spermatogonia.

When spermatogenesis advanced to P14, MILI and GASZ signals largely overlapped in spermatogonia, and co-localized entirely with each other in wildtype spermatocytes (Fig. 3C and Supplementary Fig. S3D). However, in mutant *Gasz^Δ19/Δ19^*testes, surviving spermatocytes showed few overlapping GASZ and MILI signals (Fig. 3C & Supplementary Fig. S3D). We further confirmed these results with PLA assays. We observed robust PLA signals in spermatocytes and relatively weaker signal intensity in spermatogonia from wildtype testicle sections (Fig. 3D), suggesting strong GASZ and MILI interactions in spermatocytes. By contrast, no PLA signals were observed in *Gasz^Δ19/Δ19^*testes (Fig. 3D), proving that the GASZ–MILI interaction was disrupted in the *Gasz^Δ19/Δ19^*germ cells by removing the *N*-terminal 19 aa from GASZ proteins.

### Disrupted GASZ–MILI interaction impairs piRNA biogenesis

**Figure 4.**
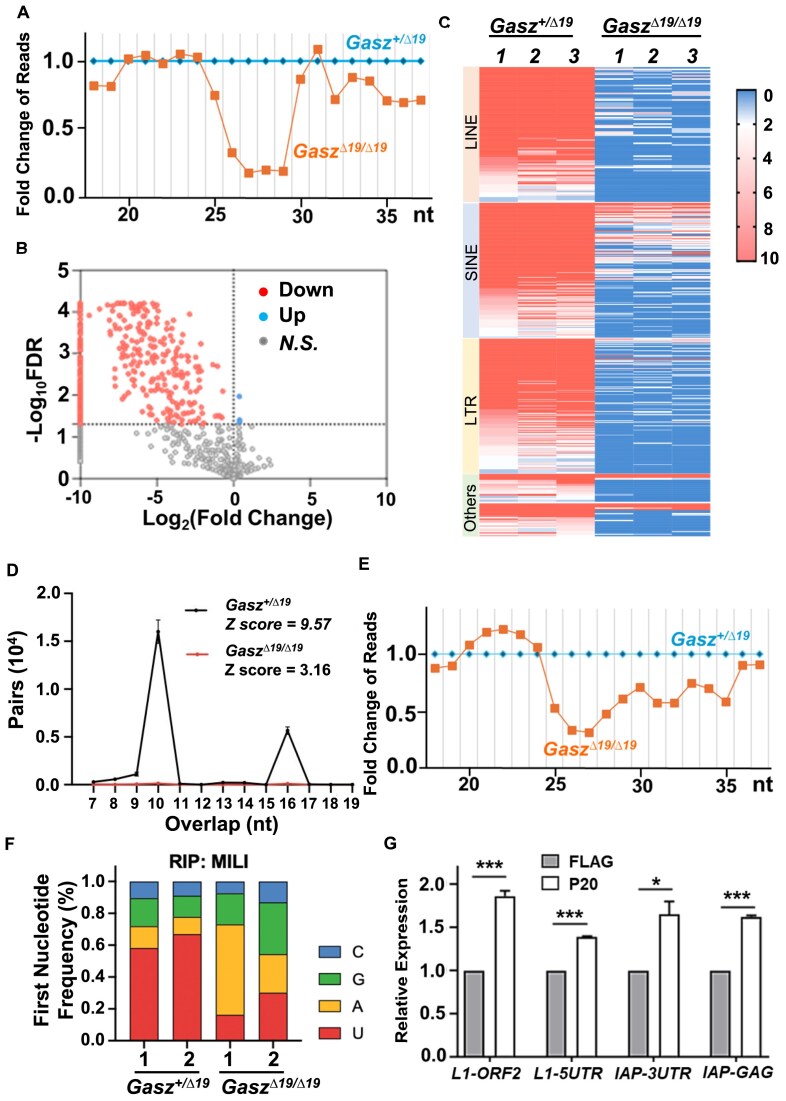
Disrupting GASZ–MILI interaction leads to reduced pre-pachytene piRNA biogenesis. (**A**) The fold changes in the reads of small RNAs from *Gasz^Δ19/Δ19^* neonatal testes versus their heterozygote littermate controls were plotted along their length (nucleotide/nt), extracted from small RNA-sequencing data. (**B**) Volcano plot of fold changes in the levels of piRNAs from P0 *Gasz^Δ19/Δ19^* versus their heterozygote littermate controls. Down: downregulated in *Gasz^Δ19/Δ19^*; Up: upregulated; *N.S.:* not significant, with *P* value > 0.05. (**C**) Heatmap to visualize all differentially expressed piRNAs with *P* value < 0.05 from P0 *Gasz^Δ19/Δ19^* and their heterozygote littermate. piRNA were grouped according to their genomic regions. Color indicates abundance of each piRNA with red color as the highest level. Each genotype group has three biological replicates in small RNA-sequencing. (**D**) Ping-pong Z score with 10 nt overlap in piRNA biogenesis from P0 *Gasz^Δ19/Δ19^* and their heterozygote testes was calculated based on three biological replicates per group. (**E**) The fold changes in reads of small RNAs from *Gasz^Δ19/Δ19^* neonatal testes *vs*. littermate controls were plotted along their length, based on small RNA-sequencing from RIP assays. Two replicates per genotype were used. (**F**) The frequencies of the first nucleotide detected in piRNAs pulled down by a MILI antibody from *Gasz^Δ19/Δ19^* neonatal testes versus littermate controls. (**G**) FLAG control peptides or FLAG-tagged P20 (the first 20 amino acids of GASZ) were introduced into primary spermatogonia by lentiviral infection, followed by real-time RT-PCR to assess the expression of transposable elements. Data were presented as mean ± SEM of three replicates; *: *P*< 0.05; ***: *P*< 0.001.

To examine whether piRNA biogenesis is affected by disrupted GASZ–MILI interaction, we collected testis from neonatal mice and conducted small RNA sequencing. We found that compared to controls, the reads of small RNAs from 25 to 29 nt, the piRNA fraction predominantly bound by MILI, significantly decreased in *Gasz^Δ19/Δ19^* (Fig. 4A and Supplementary Fig. S4A). In addition, most piRNAs were downregulated in *Gasz^Δ19/Δ19^* testes (Fig. 4B and C and Supplementary Table S1), indicating impaired piRNA biogenesis. In prospermatogonia, more than half of piRNAs are derived from transposable elements and in turn repress transposon expression [9, 18]. We thus analyzed the genomic loci of piRNAs that were differentially expressed between *Gasz^+/Δ19^* and *Gasz^Δ19/Δ19^* testes, using online databases [40]. We found that the majority of these piRNAs were indeed from transposable elements, including LINEs, SINEs, and LTRs (Fig. 4C). A relatively small number of differentially expressed piRNAs were from other genomic regions such as exon-coding sequences (Fig. 4C), in line with published reports [9, 18]. Consistent with these findings, the transposon expression was upregulated in *Gasz^Δ19/Δ19^* testes (Supplementary Fig. S4B). We further found that a substantial fraction of piRNAs detected contained uridine at their 5′ end, a hallmark characteristic of piRNAs that are generated from the primary pathway [37]. This fraction was decreased in *Gasz^Δ19/Δ19^* testes (Supplementary Fig. S4C), suggesting disrupted GASZ–MILI interaction impaired primary piRNA generation. During piRNA biogenesis, primary piRNAs guide the cleavage of complementary transposon sequences to generate new piRNAs that can be subsequently amplified in the so-called ping-pong feedback loop. To assess whether disrupted GASZ–MILI interaction also impacted the secondary piRNA generation, the *Z*-score of ping-pong cycle was calculated by comparing the frequency of piRNA pairs with a specific 10-nucleotide overlap (representing piRNAs from the ping-pong cycle) to that from background overlap distribution [38]. We found that Z-score was much lower in *Gasz^Δ19/Δ19^* groups compared to controls (Fig. 4D), indicating that ping-pong reaction was also negatively affected by the disrupted GASZ–MILI interaction.

We further conducted RNA immunoprecipitation (RIP) with a MILI antibody on *Gasz^+/Δ19^* and *Gasz^Δ19/Δ19^* neonatal testes. Similarly, we observed that small RNAs from 25 to 29 nt were drastically downregulated in *Gasz^Δ19/Δ19^* testes (Fig. 4E and Supplementary Fig. S4D). The overall abundance of piRNAs that were pulled down by MILI decreased in *Gasz^Δ19/Δ19^* testis (Supplementary Fig. S4E and F & Supplementary Table S2). In addition, the uridine preference at the first nucleotide of piRNAs diminished in *Gasz^Δ19/Δ19^* samples (Fig. 4F).

Because the whole testis contains both germ cells and somatic cells, to directly assess the effect of disrupted GASZ–MILI interaction on germ cells, we introduced a FLAG-tagged peptide (P20) containing the first 20 aa of GASZ into primarily cultured wildtype spermatogonia by lentiviral infection. As described in Fig. 2E, this peptide reduced GASZ–MILI interactions by competition. We then conducted small RNA-sequencing and real-time RT-PCR analyses on P20-expressing spermatogonia and the controls with FLAG peptide expression. We observed that piRNA expression was similarly reduced in P20-expressing spermatogonia, compared to controls (Supplementary Fig. S4G and H & Supplementary Table S3). Consequently, the levels of transposable elements were elevated in P20 groups (Fig. 4G & Supplementary Fig. S4I). Taken together, our data revealed that disrupting GASZ–MILI interaction reduced piRNA biogenesis in spermatogonia and released the inhibition of transposon expression from piRNA inhibition.

### Disrupted GASZ–MILI interaction during the embryonic stage affects both spermatogonia and spermatocyte development

In mice, prospermatogonia develop from bi-potential primordial germ cells during the embryonic stage, and then become spermatogonia after birth [6]. The first wave of spermatogenesis occurs during the first 6-week postnatal development. A small fraction of undifferentiated spermatogonia remain as SSCs that support steady-state spermatogenesis in adulthood [6]. Spermatogonia and SSCs are located at the basal compartment, while spermatocytes and spermatids move toward the center (*i.e*. adluminal compartment) of seminiferous tubules. Spermatocytes from the first wave of spermatogenesis can be detected before P14, while haploid spermatids form at P20.

**Figure 5.**
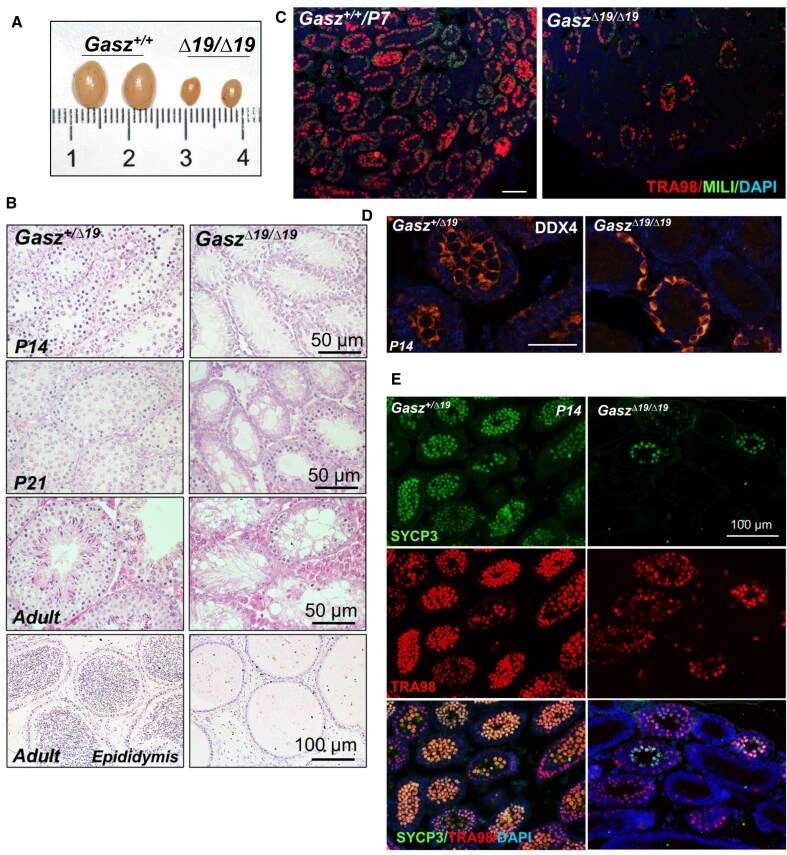
Disrupting GASZ–MILI interaction in embryonic germ cells leads to reduced formation of spermatogonia and spermatocytes. (**A**) The morphology of testes from *Gasz^Δ19/Δ19^* adult mice and wildtype littermate controls. (**B**) Histological studies on testis or epididymis sections from *Gasz^Δ19/Δ19^*mice and littermate controls at P14, P21, and adulthood. (**C**) IHF performed on testes from P7 mice with antibodies against TRA98 and MILI. (**D**) IHF performed on testes from P14 mice with an antibody against DDX4. (**C** and **D**) Scale bar: 50 μm. (**E**) IHF performed on testes from P14 mice with antibodies against TRA98 and SYCP3. (**C–E**) All tissue sections were counterstained with DAPI.

Compared to wildtype mice, both heterozygote male GASZ mutant and homozygote female GASZ mutant mice were fertile (Supplementary Fig. S5A). By contrast, we observed that the testes of homozygote GASZ mutants became significantly smaller in adulthood compared to wildtype littermate controls (Fig. 5A), indicating reduced spermatogenesis. We thus examined the germ cell development of these mice at different ages using histology studies (Fig. 5B & Supplementary Fig. S5B). We found significantly reduced spermatocyte formation in homozygote GASZ mutants at P14 (Fig. 5B). Few round spermatids were detected at P21 and in adult *Gasz^Δ19/Δ19^*testes, and no sperm were found in the epididymis (Fig. 5B). Consistently, male *Gasz^Δ19/Δ19^* mice were infertile (Supplementary Fig. S5A).

The germ cell composition of these mice was further scrutinized using IHF to detect DDX4+ (germ cells from prospermatogonia to spermatids), SYCP3+ (spermatocytes), or PNA+ (spermatids) cells. We found no evident difference in DDX4+, GASZ+, or MILI+ prospermatogonia formation between homozygote GASZ mutants and littermate controls at P0 (Supplementary Fig. S5C), indicating that prospermatogonial development was not significantly affected. Although the seminiferous tubules between wildtype and *Gasz* homozygote mutant at P7 appeared to be histologically similar (Supplementary Fig. S5B), IHF assays showed significantly decreased DDX4, GASZ, and MILI signals at P7 (Supplementary Fig. S4D). To examine whether the decreased staining of these IMC proteins was due to a reduction of the overall germ cell population, we performed IHF with TRA98, a pan-germ cell marker. We found the TRA98 signal was also decreased at P7 (Fig. 5C), suggesting the whole spermatogonial population was impaired. At P14, compared to well-formed DDX4 + spermatocytes in the middle of seminiferous tubules from control testes, we observed that DDX4 + germ cells mainly located in the basal compartment in *Gasz^Δ19/Δ19^*testes where spermatogonia resided (Fig. 5D). Indeed, significantly reduced SYCP3 + spermatocytes from P14 *Gasz^Δ19/Δ19^* mice were detected by IHF (Fig. 5E). Only less than 10% testis sections contained SYCP3 + spermatocytes (Fig. 5E). When small dot-like PNA + proacrosomal structures were readily detected in round spermatids from P21 control mice, only residual PNA signals were observed in *Gasz^Δ19/Δ19^* seminiferous tubules (Supplementary Fig. S5E). No PNA + spermatids were observed in adult homozygote mutant mice (Supplementary Fig. S5E). These data revealed that both spermatogonia and spermatocytes were impaired by disrupting GASZ–MILI interaction during the embryonic stage. A few remaining spermatocytes in *Gasz^Δ19/Δ19^* mice were unable to properly develop into round spermatids.

### Blocking GASZ–MILI interaction in adult mice abolishes spermatid formation

**Figure 6.**
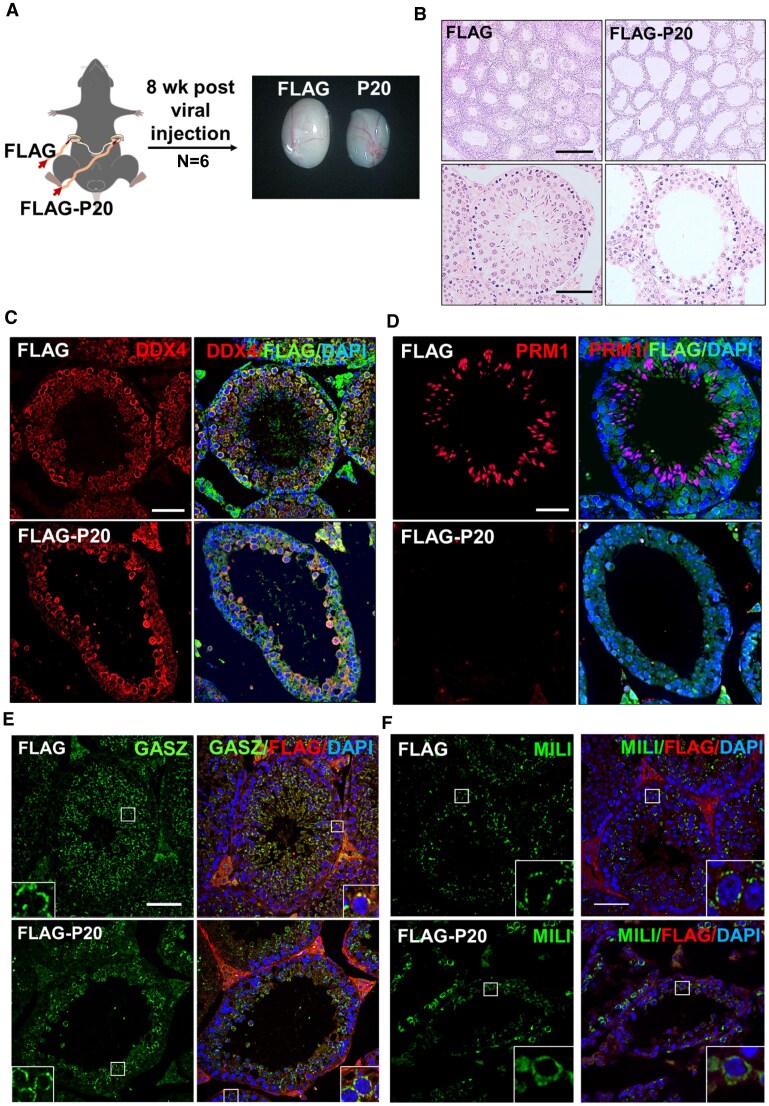
Disrupting GASZ–MILI interaction in adult testes leads to reduced spermatocyte and spermatid formation. (**A**) Virus expressing a FLAG control peptide or a FLAG-tagged P20 was injected into testes via efferent ducts. Testes were examined 8 week post viral injection. (**B**) Histology study on testes collected 8 weeks post viral injection of FLAG and FLAG-P20 expressing virus. Few spermatids were detected upon FLAG-P20 injection. (**C**–**F**) IHF were performed using antibodies against FLAG and DDX4 (**C**), PRM1 (**D**), GASZ (**E**), or MILI (**F**), counterstained with DAPI. (**E** and **F**) Insets are blow-ups of representative images to show the localization patterns of GASZ and MILI in germ cells 8 weeks post viral injection of FLAG control peptide and P20 expressing virus. The two left panels highlight GASZ signals in round spermatids, and the two panels on the right show GASZ signals in spermatocytes. (**B**-**F**) Scale bar: 50 μm.

To explore the functional impact of the disrupted GASZ–MILI interaction during steady-state spermatogenesis, we injected adult mouse testes with lentivirus expressing a FLAG-tagged peptide containing the *N*-terminal 20 aa (FLAG-P20) of GASZ. In the contralateral testis from the same mouse, we introduced lentivirus that only expressed FLAG peptide as a control. After 8 weeks, we observed that the testes injected with FLAG-P20, the GASZ–MILI interaction blocking peptide, were smaller compared to the control testes (Fig. 6A). Consistent with findings above with *Gasz^Δ19/Δ19^*mutants, histology studies demonstrated that spermatocyte formation was reduced, and no elongated spermatids were found in the testes with P20 injection (Fig. 6B and Supplementary Fig. S6A). We also confirmed this finding with IHF with DDX4 and PRM1 (haploid spermatid) antibodies. We found reduced DDX4 + germ cells in the adluminal compartment of seminiferous tubules when GASZ–MILI interaction was blocked by P20 peptides (Fig. 6C). Few PRM1 + spermatids were detected in the P20 groups (Fig. 6D and Supplementary Fig. S6B). In addition, we found that GASZ localization pattern was not significantly altered in the P20-injected testes (Fig. 6E). By contrast, MILI signals displayed a diffused pattern in the cytoplasm of germ cells with GASZ–MILI interaction blocking peptides (Fig. 6F and Supplementary Fig. S6C). Taken together, these data demonstrated that disrupting GASZ–MILI interaction in adulthood compromises the steady-state spermatogenesis.

## Discussion

The piRNAs are essential for male fertility by maintaining genomic stability through transposon repression. Accumulating evidence supports that primary piRNA processing occurs at IMC, a type of electron dense granules in germ cells [8, 14, 17, 26, 27]. IMC construction largely relies on two critical components: first, aggregated mitochondria that provide a subcellular compartment and energy for piRNA processing; and second, interactive proteins at mitochondria that form cement to stabilize aggregated mitochondria and coordinate each other in piRNA processing. Multiple IMC-localized proteins have been identified, including MILI, a major player in piRNA biogenesis. But MILI lacks MLS, and it remains elusive how MILI is recruited to IMC. In this study, we revealed that GASZ was the key partner in directly recruiting MILI to mitochondria within IMC in germ cells. Disrupting GASZ–MILI interaction compromised piRNA biogenesis and increased transposon expression, which in turn impaired germ cell development and led to male infertility. Our findings thus critically inform how the piRNA biogenesis machinery is constructed *via* protein interactions to preserve germline DNA integrity for proper germ cell development.

Because proteins at IMC form an interacting network, Co-IP of one protein will usually pull the others with them. Therefore, in addition to Co-IP of MILI and GASZ in testis, we demonstrated that MILI, but not other IMC proteins (*e.g*. DDX4 and MIWI), translocated from cytoplasm to mitochondria upon GASZ co-expression in somatic cells that lack other germ cell-specific proteins. These data, together with findings from PLA assays on testes and those from GASZ–MILI interaction, mutant mice strongly prove our hypothesis that GASZ functions as the direct and essential partner for MILI at IMC in germ cells. Notably, MILI can also be found in chromatoid body in late spermatocytes and spermatids, while GASZ is entirely associated with mitochondria. Based on AlphaFold 2 prediction, the first ∼20 aa residues of GASZ formed hook-like structures to loosely pull MILI into proximity. Such an interacting interface is likely crucial for MILI to localize at mitochondria within IMC and then translocate from IMC to chromatoid body. A recent study revealed that piRNA loading triggered MIWI, another PIWI family member, to move from IMC to chromatoid body [41]. It will be interesting to investigate whether a similar mechanism contributes to MILI translocation.

Published studies showed that *Mili* knockout reduced piRNA biogenesis and led to male infertility [1, 42]. In this study, we observed similar phenomena when MILI could not interact with GASZ and diffused into the cytoplasm from mitochondria. The piRNAs from transposable elements were significantly reduced in GASZ–MILI interaction mutant cells, releasing transposon from piRNA repression. Subsequently, postnatal spermatogonial development was compromised, and few spermatocytes were formed. These data confirmed the critical role of MILI in the first wave of spermatogenesis and clearly revealed that MILI partners with GASZ in fetal piRNA processing. In addition, although MILI is highly expressed in spermatocytes, deleting *Mili* from prospermatogonia or disrupting GASZ–MILI interaction during the embryonic stage abolishes spermatocyte formation in the early stage of spermatogenesis, preventing one from examining the role of MILI in germ cell development during adulthood. To overcome this limitation, we utilized a peptide that could efficiently block GASZ–MILI interaction. We found that spermatocyte formation decreased, and few spermatids formed when MILI was dislocated from mitochondria by this blocking peptide in adult mice. These data strongly support the critical role of MILI in spermatocyte and spermatid development during steady-state spermatogenesis in adulthood.

GASZ proteins self-interact with each other at mitochondria and also partner with mitofusin to aggregate mitochondria. Deleting MLS from GASZ or disrupting its self-interaction domain abolishes IMC formation [26, 43]. Although the interaction of MILI with GASZ strengthens mitochondrial clustering and stabilizes IMC, in our study, IMC partially remained when MILI was dissociated from mitochondria in GASZ mutant mice. This is different from *Gasz* knockout study, where germ cells lack IMC, and the reproductive defects were caused by the absence of GASZ proteins from the embryonic stage. In the GASZ–MILI interaction mutant mice, only the *N*-terminal aa 2–20 of GASZ protein was deleted. Mutant GASZ proteins thus could still be expressed and were able to aggregate mitochondria. These data highlight the critical roles of GASZ proteins in initiating IMC assembly by clustering mitochondria during the embryonic stage and then acting as a central anchorage for MILI and possibly other proteins at IMC. It is worth noting that the *N*-terminal aa 2–20 of GASZ residues do not constitute any known RNA/DNA binding domain. Nevertheless, the levels of piRNAs, especially the 25–29 nt fraction that bound by MILI, significantly reduced, providing solid evidence that GASZ does not directly process piRNAs, but provide essential IMC localization for MILI to function in subsequent piRNA biogenesis.

Infertility affects ∼48.5 million couples worldwide, with half due to male factors [44]. More than 30% of male infertile cases are idiopathic [44]. Substantial evidence has linked dysregulated piRNA biogenesis to impaired male fertility in both mice and humans [9, 19, 21, 24, 25, 27, 45–48]. Notably, GASZ protein sequences are highly conserved across mammalian species. So far, several human GASZ isoforms have been identified, one of which lacks the *N*-terminal domain for its interaction with MILI. Interestingly, based on the GTEx Portal, an online expression database, the expression of this isoform was significantly lower than that of the full-length GASZ proteins in testes, suggesting an evolutional disadvantage of this mutant isoform. Future investigation into various human GASZ isoforms may discover a mechanistic cause for male infertility, eventually leading to targeted therapies to improve male reproduction.

## Supplementary Material

gkaf957_Supplemental_Files

## Data Availability

Raw small RNA-seq data were uploaded to SRA repository (SUB15077364) and will be accessible to the public upon manuscript publication.
