# Supplementary material for: GASZ directly recruits MILI to the intermitochondrial cement for piRNA biogenesis and male germ cell development
Source: Nucleic Acids Res. 2025 Oct 8;53(18):gkaf957. doi: 10.1093/nar/gkaf957 (PMC12507516; doi:10.1093/nar/gkaf957)
Supplement: gkaf957_Supplemental_Files [file gkaf957_supplemental_files.zip › Supplemental figure and figure legend.pdf]

Supplemental Figure Legends

Fig. S1. GASZ directly recruits MILI but not MIWI to mitochondria. (**A**) Ds-RED-tagged RNABP9 was co-expressed with Su9 MLS-tagged GFP or EGFP-GASZ fusion proteins in HeLa cells. Protein subcellular localization was indicated by red and green fluorescence signals. Nuclei were identified by DAPI staining. (**B**) FLAG-tagged MILI was co-expressed with EGFP-GASZ fusion proteins in HeLa cells. The subcellular localization of GASZ and MILI proteins were visualized by GFP and IF using an antibody against MILI (Grey), co-stained with MitoTracker Red and counterstained with DAPI. (**C**) FLAG-tagged DDX4 or MIWI was co-expressed with either Su9 MLS-tagged GFP or EGFP-GASZ fusion proteins in HeLa cells. Protein subcellular localization was visualized by IF using antibodies against GFP and FLAG (Red), counterstained with DAPI. (**D**) FLAG-tagged MILI or MIWI was co-expressed with GFP-tagged GASZ in 293T cells. Cell lysates were immunoprecipitated (IP) with FLAG affinity beads, followed by Western Blot analyses. (**E**) HIS-tagged MILI and GST-tagged GASZ expressed from bacteria were subjected to Ni-NTA resin purification, followed by Western Blot analyses. Total proteins from bacteria lysate and the resin-eluted fraction (Elution) were visualized with Coomassie Blue staining (the left panel). Western Blots (WB) were performed on the eluted samples. (**F**) PLA assay on mouse gonad from E16.5 embryos with MILI and GASZ antibodies. Red signals indicate interaction between MILI and GASZ. The panel below shows negative control using GASZ antibody only. (**D-E**) The molecular weights (MW) of the proteins to be detected are provided on the right side of the panels. Arrows indicate the position of the protein ladders with known MW.

Fig. S2. Identify the critical domain of GASZ interacting with MILI. (**A**) GFP-tagged MILI proteins were co-expressed with FLAG-tagged full-length GASZ or various GASZ deletion mutants in 293T cells. Cell lysates were immunoprecipitated (IP) with FLAG affinity beads for Western Blot (WB) analyses. The molecular weights (MW) of the proteins to be detected are provided on the right side of the panels. Arrows indicate the position of the protein ladders with

known MW. (**B**) MILI protein structure (the upper panels) and GASZ-MILI interaction interface (the lower panels) were predicted by AlphaFold 2 in google Colab, visualized by PyMOL. The first 500 aa residues of MILI (yellow) were used for predicating its interaction with GASZ (red). All top-ranked five models showed the similar structure of MILI protein and GASZ-MILI interaction interface (the left panels). (**C**) GASZ-MILI interacting aa residues predicted by AlphaFold 2 in google Colab, visualized by PyMOL. A blow-up insert demonstrates the N-terminal ~20 aa of GASZ (yellow) interacting with MILI (grey). (**D**) FLAG or FLAG-tagged peptide expressing the first 20 aa of GASZ protein (P20) was introduced into primarily cultured spermatogonia *via* lentiviral infection. MILI and GASZ interaction was examined by Co-IP with a MILI antibody, followed by Western Blot analyses. The relative levels of GASZ proteins of input and ones co-precipitated with MILI were assessed by signal intensity that appeared on the blots. Data were presented as mean ± SEM of 3 technical replicates.

Fig. S3. Establishing a mutant GASZ mouse model with disrupted GASZ-MILI interaction. (**A**) The DNA sequences of established *Gasz*$^{\Delta 19/\Delta 19}$ founder mice were analyzed by Sanger-sequencing. Sequences encoding the 19 aa residues after ATG codon were correctly deleted from *Gasz* mutant alleles. (**B**) An example shows the results of PCR to analyze the alleles of *Gasz*$^{\Delta 19/\Delta 19}$ and wildtype mice. The mutant allele generates a 252 bp band, 57 bp shorter than the PCR product from the wildtype allele. (**C**) IHF with a TOMM20 antibody on testis sections from mice at P7. (**D**) IHF with antibodies against MILI and GASZ, counterstain with DAPI on testis sections from mice at P14. Upper panel: IHF on wildtype testes. Strong MILI and GASZ signals overlapped entirely in spermatocytes. Middle and bottom panels: IHF on *Gasz*$^{\Delta 19/\Delta 19}$ testes. A higher photo exposure was used on *Gasz*$^{\Delta 19/\Delta 19}$ than that on wildtype mouse testis in bottom panels to visualize weak subcellular signals of GASZ and MILI in spermatocytes. Inset shows blow-up images of *Gasz*$^{\Delta 19/\Delta 19}$ remaining spermatocytes, in which weak GASZ and MILI signals were not co-localized.

Fig. S4. Disrupting GASZ-MILI interaction leads to reduced piRNA biogenesis. (**A**) Size distribution of small RNAs along their length (nt), based on small RNA-sequencing data from P0 *Gasz*$^{\Delta 19/\Delta 19}$ and their littermate control testes. N=3. (**B**) Real-time RT-PCR to assess the transcript levels of transposable elements from P0 *Gasz*$^{\Delta 19/\Delta 19}$ and their littermate control testes. Three pairs of primers amplifying different *Line 1* mRNA regions were used, and all showed increased levels in *Gasz*$^{\Delta 19/\Delta 19}$ testes. (**C**) The frequences of the first nucleotide detected in piRNAs from *Gasz*$^{\Delta 19/\Delta 19}$ neonatal testes *vs.* littermate controls. (**D**) Size distribution of small RNAs along their length (nt), based on small RNA-sequencing data from MILI-immunoprecipitated P0 *Gasz*$^{\Delta 19/\Delta 19}$ and their littermate control testes. N=2. (**E**) Volcano plot on piRNAs pulled down by MILI antibodies from RIP/small RNA-seq assays on P0 *Gasz*$^{\Delta 19/\Delta 19}$ and their heterozygote littermate controls. Down: downregulated; Up: upregulated; *N.S.:* not significant, with p value > 0.05. (**F**) Heatmap to visualize piRNAs (p value < 0.05) that are pulled down by MILI antibodies from RIP/small RNA-seq assays on P0 *Gasz*$^{\Delta 19/\Delta 19}$ *vs.* their heterozygote littermates. Each genotype group has two biological replicates (Rep). (**G**) The read counts of small RNAs were plotted against their length (nt), based on small RNA-sequencing on spermatogonia expressing FLAG or FLAG-P20 (P20) peptides. Three biological replicates per group were used. (**H-I**) FLAG or P20 from the first 20 amino acids of GASZ were introduced into primary spermatogonia by lentiviral infection, followed by real-time RT-PCR to assess the expression of piRNAs (**H**) or transposable elements (**I**). (**B, H, I**) Data were presented as mean ± SEM of 3 replicates; *: p<0.05; **: p<0.01; ***: p<0.001. *N.S.:* not significant.

Fig. S5. Disrupting GASZ-MILI interaction during the embryonic stage impairs spermatogonia and spermatocyte formation. (**A**) Litters per dam generated by breeding them with *Gasz*$^{+/+}$, *Gasz*$^{+/\Delta 19}$, *Gasz*$^{\Delta 19/\Delta 19}$ male mice. *N.S.:* no significance. (**B**) Histological studies on testis sections from *Gasz*$^{+/\Delta 19}$ and *Gasz*$^{\Delta 19/\Delta 19}$ mice at P0 and P7. Scale bar: 50 μm. (**C-D**) IHF on testes from P0 (**C**) or P7 (**D**) *Gasz*$^{+/\Delta 19}$ and *Gasz*$^{\Delta 19/\Delta 19}$ mice with antibodies against DDX4, MILI,

or GASZ. (**E**) PNA staining on testes from *Gasz*$^{+/\Delta 19}$ and *Gasz*$^{\Delta 19/\Delta 19}$ mice at P21 and 3-month-old.

Fig. S6. Disrupting GASZ-MILI interaction during adulthood reduces spermatocyte and spermatid development. (**A**) Percentage of seminiferous tubule that contain less spermatocytes (one layer of spermatocytes) in FLAG vs. FLAG-P20 injected mice was calculated based on the histology of about 10 testis sections from 6 biological replicates per group. (**B**) Percentage of seminiferous tubule that contain less or no PRM1+ spermatids in FLAG vs. FLAG-P20 injected mice was calculated based on IHF of 6 testis sections from 3 biological replicates per group. (**C**) IHF were performed using antibodies against FLAG and MILI, counterstained with DAPI. Insets are blow-up of representative images to show the localization patterns of MILI in germ cells 8 weeks post viral injection of FLAG control peptide and P20 expressing virus. Scale bar: 50 μm. (**D**) FLAG-tagged MILI or GASZ were transfected into 293T cells, and Western Blots on those cells and wildtype testis were conducted using a MILI antibody or a home-made GASZ antibody. FLAG-GASZ is about 1.5 KD larger than its endogenous counterpart from testes. GASZ antibody specifically detects GASZ proteins in GASZ expressing cells and testis. (**E**) EGFP-GASZ and EGFP-GASZ without MLS were transfected into 293T, and IF was conducted using a GFP antibody and a home-made GASZ antibody, counterstained with DAPI. GASZ antibody only detects GFP+ cells.

Supplemental Tables

Supplemental Table S1. Differentially expressed piRNAs between *Gasz*$^{+/\Delta 19}$ and *Gasz*$^{\Delta 19/\Delta 19}$ neonatal testes

Supplemental Table S2. Differentially expressed piRNAs pulled down by MILI antibodies from *Gasz*$^{+/\Delta 19}$ *vs. Gasz*$^{\Delta 19/\Delta 19}$ neonatal testes

Supplemental Table S3. Differentially expressed piRNAs from cultured spermatogonia with FLAG *vs.* P20 peptide expression

Supplemental Table S4. Sequences of oligonucleotides used in this study

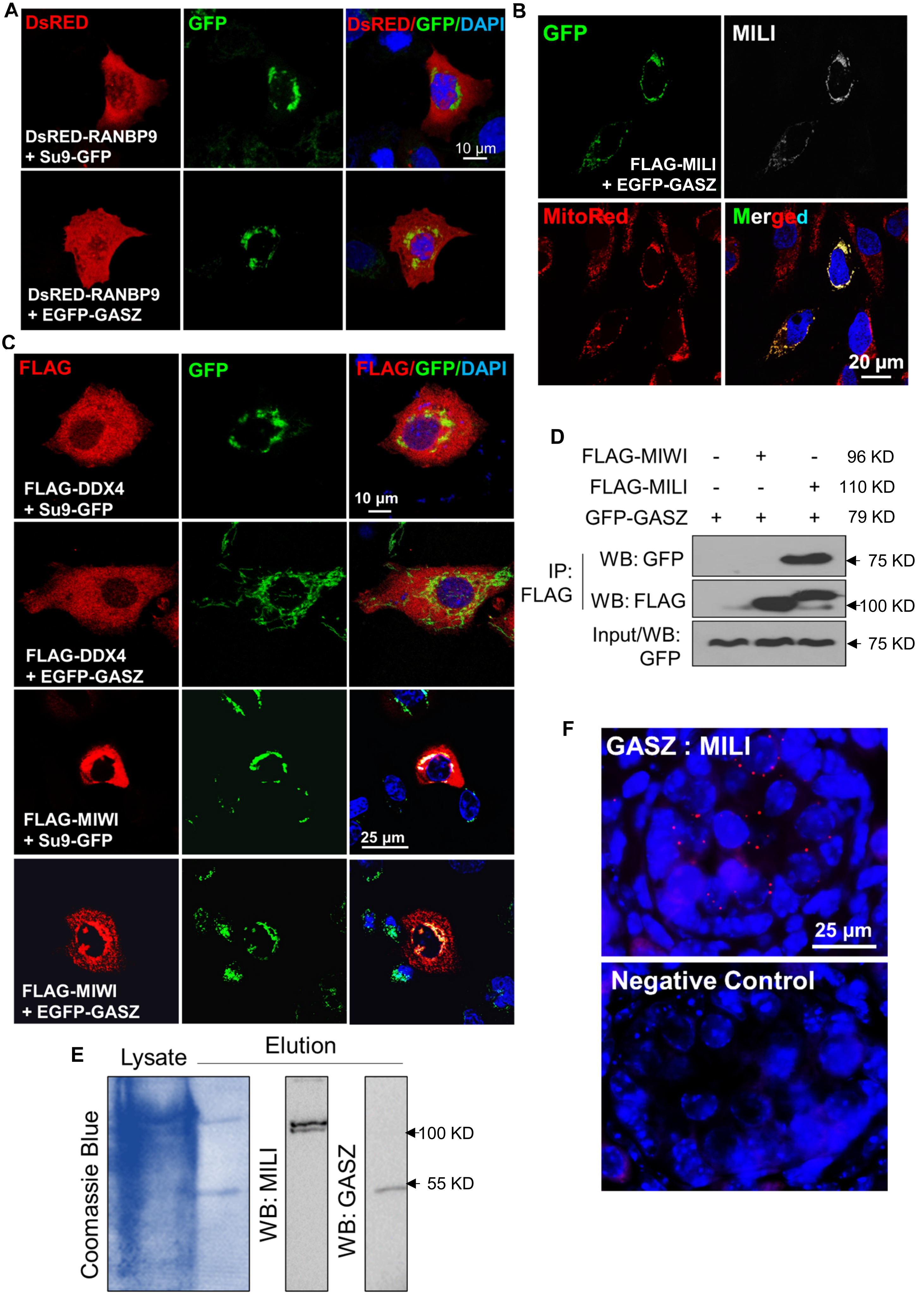

Miao et al., Figure S1

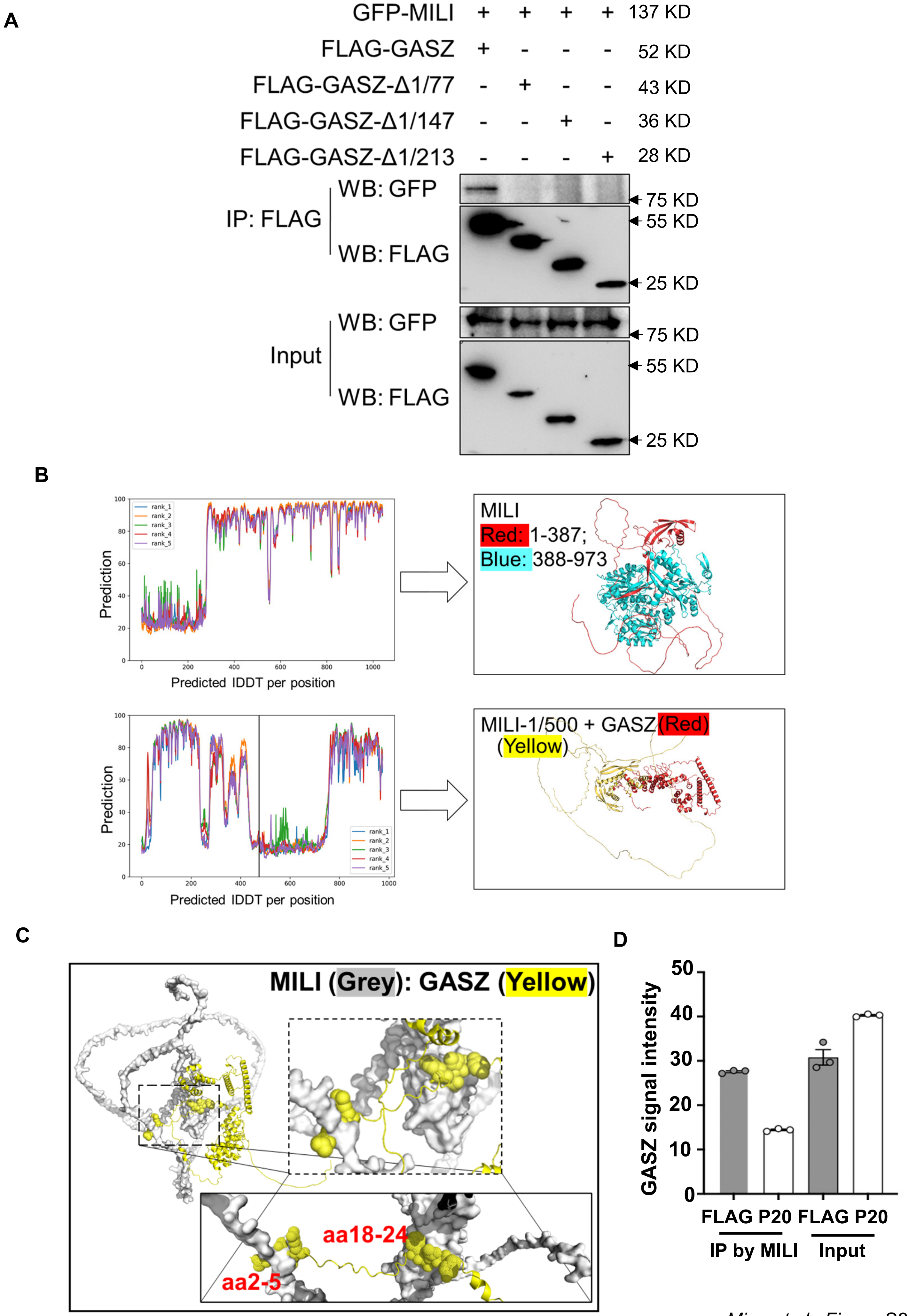

Miao et al., Figure S2

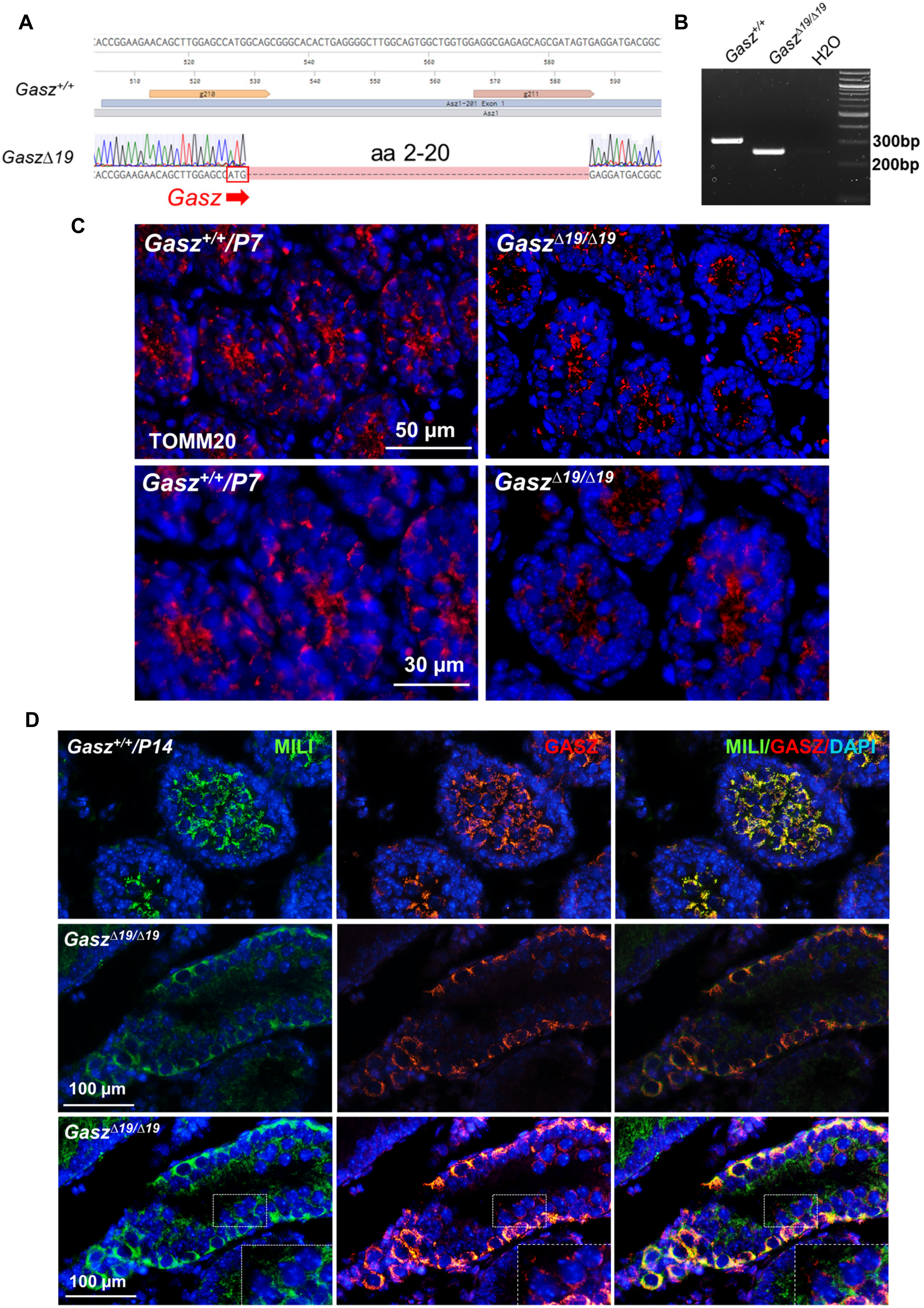

Miao et al., Figure S3

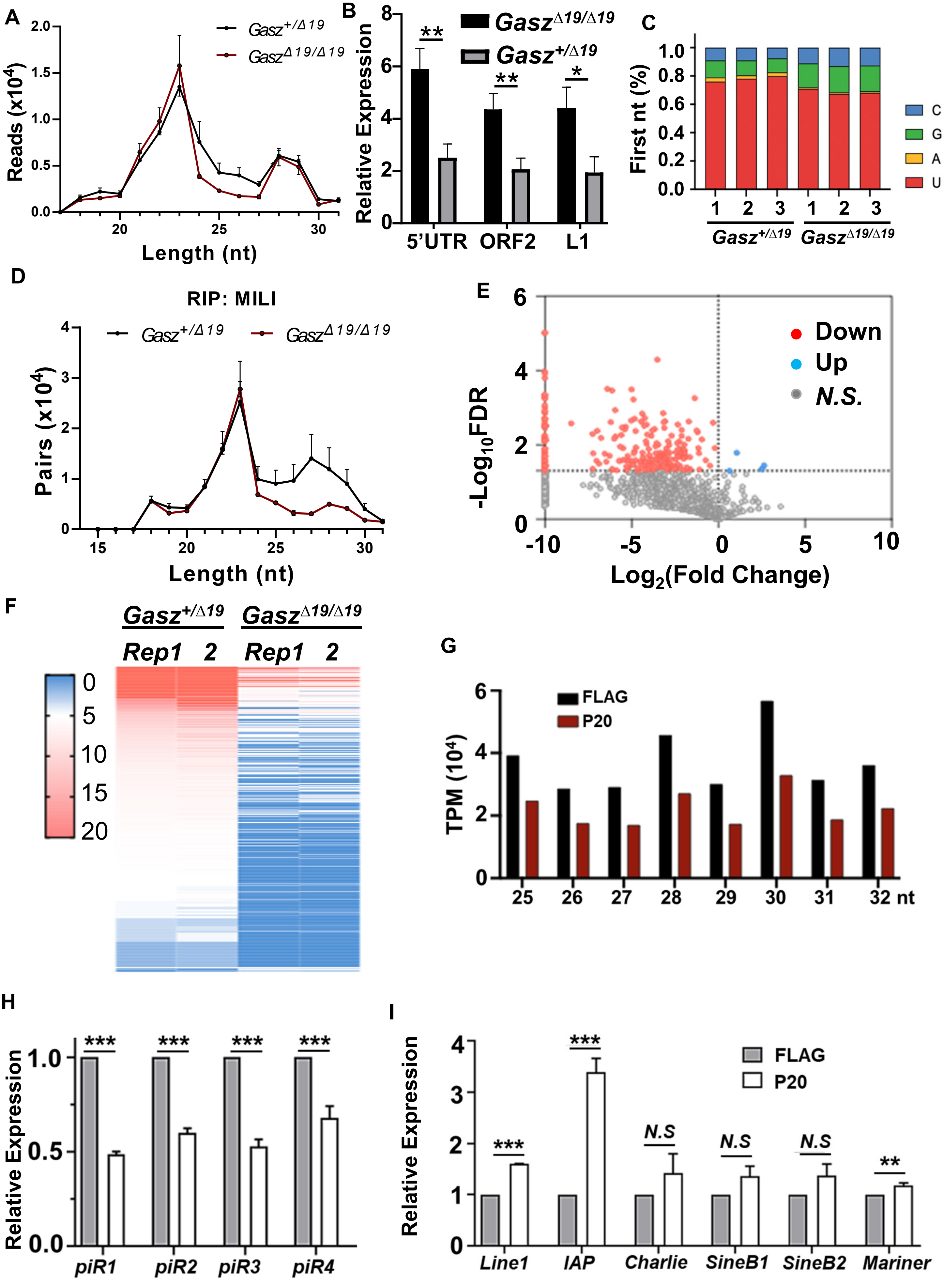

Miao et al., Figure S4

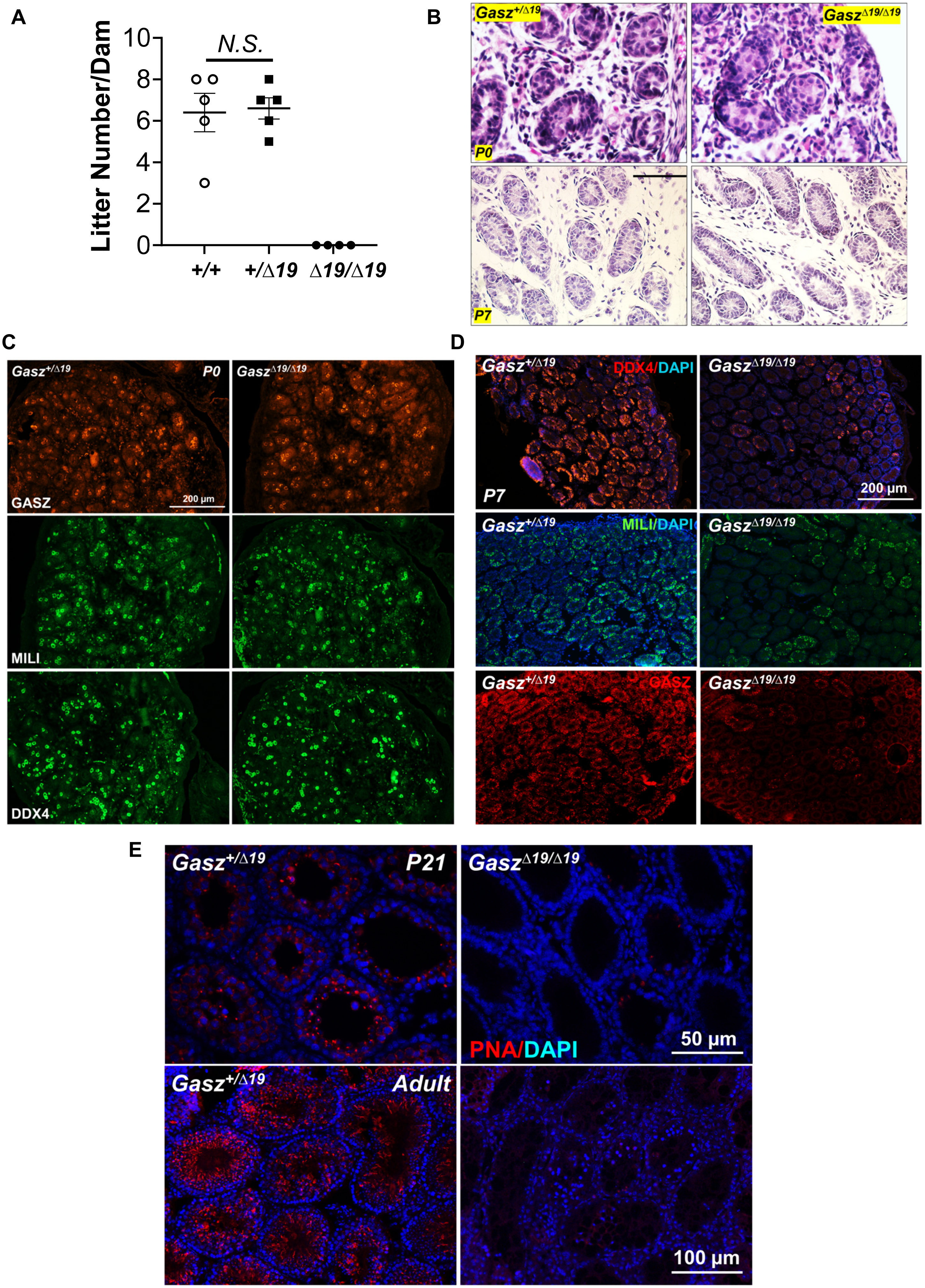

Miao et al., Figure S5

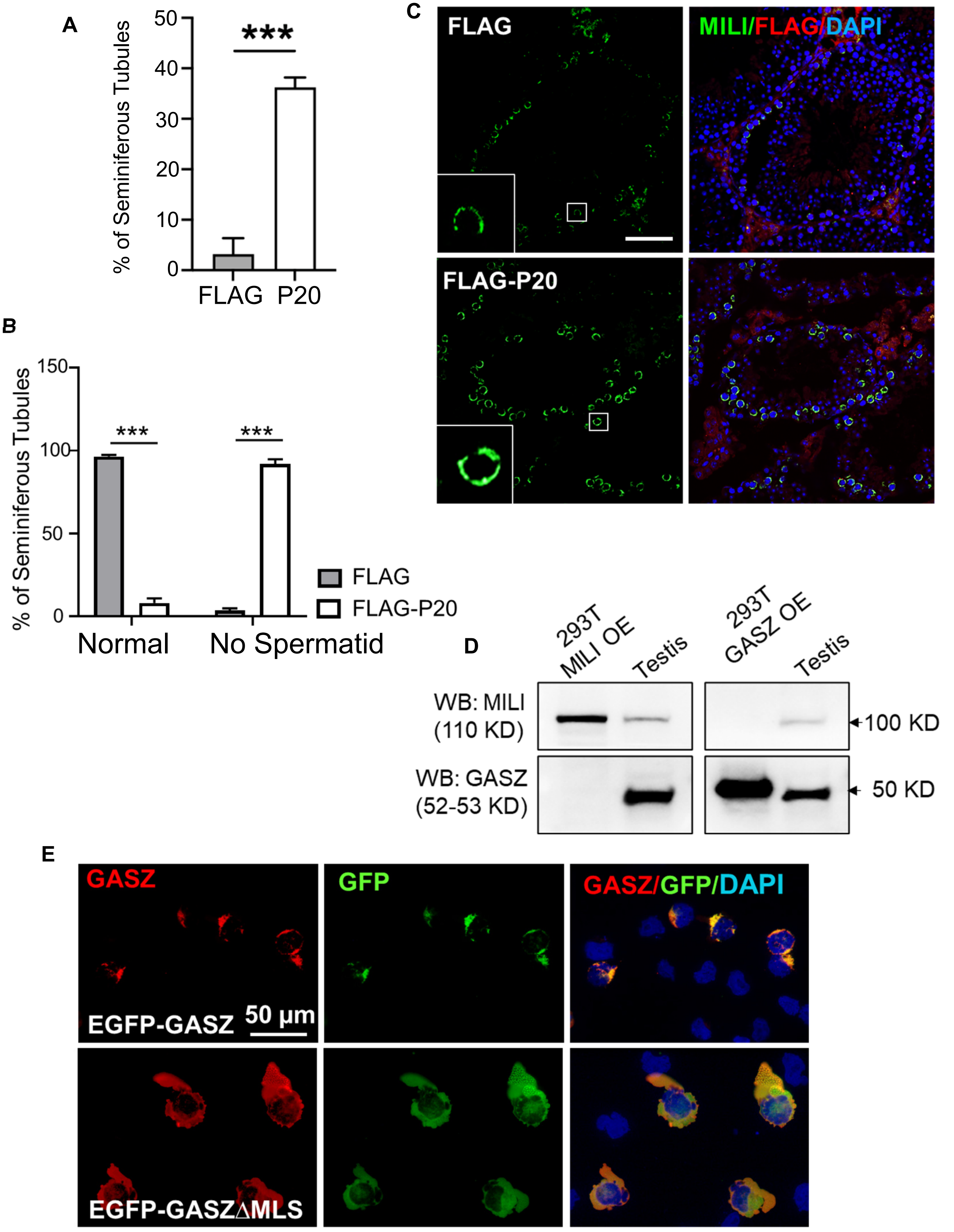

Miao et al., Figure S6
